# BSDR: A Data-Efficient Deep Learning-Based Hyperspectral Band Selection Algorithm Using Discrete Relaxation

**DOI:** 10.3390/s24237771

**Published:** 2024-12-04

**Authors:** Mohammad Rahman, Shyh Wei Teng, Manzur Murshed, Manoranjan Paul, David Brennan

**Affiliations:** 1 Institute of Innovation, Science and Sustainability, Federation University Australia, University Drive, Mt Helen, VIC 3350, Australia; shyh.wei.teng@federation.edu.au; 2 Cooperative Research Centre for High Performance Soils, Callaghan, NSW 2308, Australia; 3 School of Information Technology, Deakin University, 221 Burwood Hwy, Burwood, VIC 3125, Australia; m.murshed@deakin.edu.au; 4 School of Computing, Mathematics and Engineering, Charles Sturt University, Panorama Ave, Bathurst, NSW 2795, Australia; mpaul@csu.edu.au; 5 Wimmera Catchment Management Authority, 24 Darlot St, Horsham, VIC 3400, Australia; david.brennan@wcma.vic.gov.au

**Keywords:** band selection, discrete relaxation, gradient-based search, hyperspectral, data-efficient

## Abstract

Hyperspectral band selection algorithms are crucial for processing high-dimensional data, which enables dimensionality reduction, improves data analysis, and enhances computational efficiency. Among these, attention-based algorithms have gained prominence by ranking bands based on their discriminative capability. However, they require a large number of model parameters, which increases the need for extensive training data. To address this challenge, we propose Band Selection through Discrete Relaxation (BSDR), a novel deep learning-based algorithm. BSDR reduces the number of learnable parameters by focusing solely on the target bands, which are typically far fewer than the original bands, thus resulting in a data-efficient configuration that minimizes training data requirements and reduces training time. The algorithm employs discrete relaxation, transforming the discrete problem of band selection into a continuous optimization task, which enables gradient-based search across the spectral dimension. Through extensive evaluations on three benchmark datasets with varying spectral dimensions and characteristics, BSDR demonstrates superior performance for both regression and classification tasks, achieving up to 25% and 34.6% improvements in overall accuracy, compared to the latest attention-based and traditional algorithms, respectively, while reducing execution time by 96.8% and 97.18%. These findings highlight BSDR’s effectiveness in addressing key challenges in hyperspectral band selection.

## 1. Introduction

Hyperspectral data are high dimensional and comprise hundreds or even thousands of spectral bands. In various fields, including agriculture [1], environmental monitoring [2], mineralogy [3], health [4], and defense [5], obtaining a low-dimensional representation of high-dimensional hyperspectral data is important for purposes such as enhancing computational efficiency and identifying the most relevant features for specific tasks [6]. This dimensionality reduction is often accomplished using hyperspectral band selection algorithms, a type of feature selection technique [7] that removes highly correlated bands and selects a subset with greater discriminative power.

Hyperspectral band selection focuses on retaining the most informative spectral bands and preserving critical patterns and features in the data while enabling efficient processing and improving task performance across diverse applications such as land cover classification [8], soil property estimation [9], crop classification [10], target detection [11], and fault diagnosis in mechanical systems [12]. While traditional band selection methods, such as the Monte Carlo Uninformative Variable Elimination (MCUVE) [13] and Successive Projections Algorithm (SPA) [14], rely on statistical metrics or heuristic approaches to select bands, modern approaches often employ advanced deep learning (DL) techniques to model complex dependencies and non-linear relationships within hyperspectral data [15]. Among these, attention-based algorithms [15,16,17,18,19,20,21,22] have gained prominence in recent years.

In DL, attention [23] is a neural network construct that assigns varying importance to different parts of the input, which allows the model to focus on relevant features for a given task. For band selection, this approach first appeared in BS-Nets [16,17], referred to as the Band Attention Module and Band Attention Network, respectively. In both cases, these modules generate weights for individual bands, based on which a number of top-ranked bands, corresponding to the given target size (*t*), are selected. This strategy has inspired several band selection algorithms [15,18,19,20,21,22], as well as other machine learning tasks [24,25,26,27].

**Figure 1:**
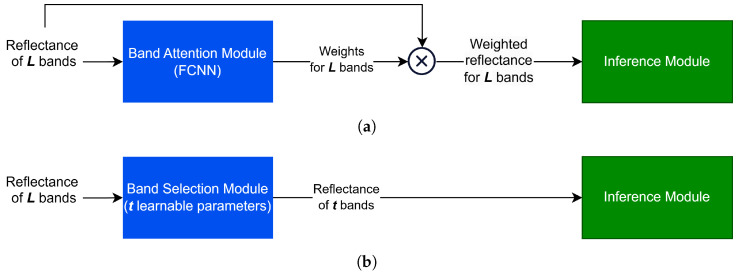
High-level architecture of the following: (**a**) Attention-based band selection algorithms; (**b**) The proposed algorithm. (**a**) Attention-based band selection algorithms: the number of model parameters depends on the original number of bands (*L*). (**b**) Proposed algorithm: the number of model parameters depends on the target size (*t*) and is independent of the original number of bands (*L*), as only the reflectance values of the *t* selected bands are processed during each training iteration.

A general architecture of attention-based band selection algorithms is depicted in Figure 1a. The Band Attention Module is a neural block—such as an FCNN, as seen in BS-Net-FC, which is one of the models under BS-Nets [16]—with an appropriate activation function at the last layer. Once the Band Attention Module generates a weight vector from the reflectance values of all bands (*L*, the total number of bands), a weighted reflectance vector is obtained through the element-wise multiplication of the reflectance vector and the weight vector. This weighted reflectance vector is then passed through another neural block, referred to here as the Inference Module, to assess the efficacy of the assigned weights through a downstream task. For instance, in BS-Nets, an autoencoder is used for unsupervised band selection, aiming to reconstruct the original hyperspectral data. In contrast, the model proposed by Zhang et al. [15], referred to hereafter as BS-Net-Classifier, employs a 1D convolutional neural network (1D-CNN) to classify maize seed varieties, selecting bands particularly relevant to this task. Some band selection algorithms inspired by BS-Nets incorporate advanced mechanisms beyond the general architecture depicted in Figure 1a to enhance band ranking. For instance, BSFormer [22] employs a transformer-based model as the Band Attention Module instead of an FCNN, while BS-Net-Classifier introduces a sparse constraint module to zero out the weights of unimportant bands below a specified threshold.

As shown in Figure 1a, the input layer of the Band Attention Module must contain *L* nodes to attend to each individual band. Similarly, the output layer, which represents the weight vector of size *L*, must also have *L* nodes. Consequently, the number of learnable model parameters scales with *L*, regardless of the specific architecture of the Band Attention Module, whether it is an FCNN, CNN, or transformer-based model. The Inference Module is similarly affected as its input is the weighted reflectance vector of size *L*. Since *L* is often large, this imposes significant computational demands and requires large training datasets, which are frequently scarce. Learning an excessive number of parameters from limited data can lead to the Hughes phenomenon, resulting in overfitting [28].

Through the effective use of model parameters for enhanced data efficiency as indicated by the ability to achieve strong generalization performance with the available training data, the number of learnable model parameters should be decoupled from the spectral dimension (*L*). To achieve this, we propose a novel band selection algorithm that focuses on selecting a subset of bands matching the target size (*t*) at each training iteration, ensuring that the number of required model parameters depends only on *t* rather than *L*. Figure 1b illustrates the high-level architecture of the proposed algorithm. As shown, the Band Selection Module includes only *t* learnable parameters, which represent the selected band indices. The module updates these parameters to capture the combination of important bands based on their relevance to the objective task, as evaluated by the Inference Module. Unlike attention-based algorithms, the proposed approach only feeds the reflectance values of the *t* selected bands into the Inference Module at each training iteration, thereby reducing the number of parameters in both the Band Selection Module and the Inference Module. This makes the algorithm inherently data-efficient, enabling effective training with a modest number of samples. The model is trained end-to-end, jointly optimizing the parameters of both modules through a unified optimization process.

While subset selection problems are generally NP-hard for optimal solutions [29], leveraging the quasi-continuous nature of hyperspectral data [30], where small changes in band index result in smooth changes in band values, allows us to apply gradient-based methods for a practical solution, which makes the subset selection task tractable. The primary challenge, however, is that band indices are discrete integers, which complicates the application of the gradient-based search methods that rely on a continuous search space. A common approach for addressing such challenges is discrete relaxation, an optimization technique that approximates discrete variables with continuous ones, enabling the use of continuous optimization methods [31]. By relaxing discrete constraints, the problem becomes differentiable, allowing for an efficient exploration of the solution space. Once optimized, the continuous values are mapped back to discrete ones to find a feasible solution to the original problem, making otherwise intractable problems solvable. In our proposed algorithm, we allow target indices to adopt non-integer values by applying discrete relaxation, which transforms the task into a continuous optimization problem suitable for gradient-based search. The rounding process has a minimal impact on the results since neighboring bands tend to be highly correlated [32]. This approach enables the solving of the problem in polynomial time with respect to the number of target indices, after which the indices are rounded to the nearest integer. Based on this, we name the proposed algorithm: Band Selection through Discrete Relaxation (BSDR).

Selecting a random subset and updating it based on its impact in predicting a target variable requires a subset selection mechanism. This mechanism must handle set inputs with properties such as permutation invariance and element uniqueness. We approximate the subset selection task using a learning mechanism that operates on vector inputs, where the vector represents a set. To ensure element uniqueness, the parameters are initialized by linearly spacing them across the spectral dimension.

We attempt to assess the efficacy of BSDR for two types of tasks: regression and classification. Since 3D spectral–spatial datasets for regression are limited, we focus on single-pixel samples. Our aim is to compare BSDR’s performance against BS-Net-FC [16] and BS-Net-Classifier [15]. Additionally, we include two traditional band selection algorithms: MCUVE [13] and SPA [14].

To sum up, the main contributions of this paper are as follows:

1. To the best of our knowledge, BSDR is the first hyperspectral band selection algorithm that applies discrete relaxation to band indices, enabling gradient-based optimization for band selection, thereby transforming the otherwise NP-hard subset selection problem into a tractable one for practical solutions.
2. Unlike modern the DL-based band selection algorithms that analyze the entire spectrum, BSDR only evaluates a limited number of bands at any given time. This approach requires significantly fewer model parameters than other DL-based models, making it not only inherently data-efficient but also enabling noticeable time efficiency without compromising predictive accuracy.
3. To the best of our knowledge, BSDR is the first band selection algorithm that enables the application of gradient-based search to a subset selection task by structuring a vector to emulate a set.

The remainder of the paper is structured as follows: Section 2 describes in detail our test datasets and the proposed algorithm. The results and relevant discussions are presented in Section 3 and Section 4, respectively. Finally, Section 5 concludes the research and highlights future directions.

## 2. Materials and Methods

### 2.1. Test Datasets

To assess the efficacy of BSDR across datasets with diverse characteristics, including variations in sample size, and number of bands, we employed three different datasets in this study. For the classification task, we utilized the Global Hyperspectral Imaging Spectral-library of Agricultural crops for Conterminous United States (GHISACONUS) [33] and Indian Pines [34] datasets. For the regression task and to demonstrate the efficacy of BSDR on hyperspectral data with a very high spectral dimension, we utilized the Land Use/Cover Area frame Survey (LUCAS) [35] dataset. In the classification task, the goal is to predict land cover classifications for the Indian Pines dataset and crop identification for the GHISACONUS dataset, while in the regression task, our aim is to estimate the Soil Organic Carbon (SOC) content. A brief overview of these datasets is provided below.

**GHISACONUS**: GHISACONUS is formed through a collaboration between the United States Geological Survey and global partners, compiling hyperspectral signatures from major agricultural crops within the Conterminous United States. Data was acquired using the Earth Observing-1 (EO-1) Hyperion hyperspectral data and supported by the USDA Cropland Data Layer as reference data. It utilizes Earth Observing-1 (EO-1) Hyperion data, which spans 220 bands with a spectral range of 400 nm to 2500 nm; however, 198 bands ranging from 437 nm to 2395 nm are available in the dataset. This dataset includes 6988 samples of five different crops, collected from seven distinct agroecological zones.

**Indian Pines**: The Indian Pines dataset captured by the Airborne Visible/Infrared Imaging Spectrometer (AVIRIS) sensor over Northwestern Indiana consists of 224 spectral bands. It primarily includes agricultural land and forest or natural vegetation. The ground truth for this dataset comprises 16 different classes, such as alfalfa, corn, grass-pasture, soybeans, wheat, woods, and other land cover types. The dataset is widely used for testing classification algorithms in hyperspectral imaging, given its challenging nature due to the high overlap in spectral signatures between some of the classes. The data cover wavelengths from 400 nm to 2500 nm, with 24 bands removed due to water absorption.

**LUCAS**: The LUCAS dataset is a large-scale, systematic survey conducted by Eurostat to monitor land use, land cover, and environmental conditions across the European Union. Launched in 2001, it gathers harmonized data to track changes in land cover over time, supporting policies related to agriculture, the environment, and climate. Through extensive field surveys, trained surveyors collect data on various land use types, soil characteristics, and vegetation from sample points across the EU. The dataset includes over 20,000 topsoil samples collected from 25 European countries, providing absorbance measurements in both the visible and near-infrared and short-wave infrared ranges, covering 400 nm to 2500 nm with a 0.5 nm spectral resolution, comprising 4200 bands.

To use these three datasets in our study, certain preprocessing steps are required. Among the 198 bands of the GHISACONUS dataset, we excluded 67 bands as information is missing for most of the samples for those bands. While the Indian Pines dataset originally provided 3D spectral–spatial data, Cai et al. [16] transformed it into single-pixel hyperspectral data to utilize it with BS-Net-FC. We have adopted the same transformation methodology in our study, excluding the background pixels. For LUCAS, we converted absorbance values into reflectance using the equation discussed in [36]:

(1) $$reflectance=\frac{1}{10^{absorbance}}.$$

Table 1 provides an overview of the three datasets. The data collection includes diverse geographic regions, encompassing a wide range of soil types with significant variability for both the Ghisaconus [33] and LUCAS [35] datasets. Additionally, the Indian Pines dataset exhibits a notable class imbalance [34], which poses unique challenges for classification tasks. Consistent performance by a band selection algorithm across these datasets would underscore its overall efficacy, demonstrating its capability to link spectral characteristics to their corresponding classes while effectively addressing complexities such as geographic diversity, soil variability, and class imbalance.

### 2.2. Proposed Algorithm

#### 2.2.1. Mathematical Foundation

Hyperspectral data for *N* samples with *L* bands can be expressed as *N* spectral vectors $v_{i}$, where $v_{i}\left(j\right)$ represents the reflectance value of the *j*-th band of the *i*-th sample, for i∈{1,2,…,N} and j∈{1,2,…,L}. To estimate a target variable $y_{i}$ for i∈{1,2,…,N}, we determine an optimal combination of *t* bands, which requires deriving the subset that comprises the indices of the selected bands.

Selecting any *t* bands yields a subset *s* such that:

(2) $$s=\{k_{1},k_{2},\ldots ,k_{t}\}$$

where $k_{p}$ represents a selected band index for all p∈1,2,…,t. Considering $f_{\theta }\left(\;\cdot \;\right)$ approximates the target variable based on the reflectance values of the selected bands in *s*, where the function *f* is parameterized on θ, the set of learnable model parameters, the prediction for the *i*-th sample $\hat{y_{i}}$ is as follows:

(3) $$\hat{y_{i}}=f_{\theta }\left(\left\{v_{i}\left(k_{1}\right),v_{i}\left(k_{2}\right),\ldots ,v_{i}\left(k_{t}\right)\right\}\right).$$

Given that $\epsilon_{i}$ represents the error term in the prediction for the *i*-th sample,

(4) $$y_{i}=\hat{y_{i}}+\epsilon_{i}\;.$$

The overall error ϵ can be minimized by minimizing the following expression:

(5) $$\begin{array}{cc} \epsilon & =\frac{1}{N}\sum_{i=1}^{N}{\left(y_{i}-\hat{y_{i}}\right)}^{2} \end{array}$$

(6) $$\begin{array}{cc} \; & =\frac{1}{N}\sum_{i=1}^{N}{\left(y_{i}-f_{\theta }\left(\left\{v_{i}\left(k_{1}\right),v_{i}\left(k_{2}\right),\ldots ,v_{i}\left(k_{t}\right)\right\}\right)\right)}^{2}. \end{array}$$

The discrete optimization problem of finding the optimal subset that minimizes ϵ can be formulated as the following objective function:

(7) $$\underset{\theta ,\{k_{1},k_{2},\ldots ,k_{t}\}}{\arg\;\min}\frac{1}{N}\sum_{i=1}^{N}{\left(y_{i}-f_{\theta }\left(\left\{v_{i}\left(k_{1}\right),v_{i}\left(k_{2}\right),\ldots ,v_{i}\left(k_{t}\right)\right\}\right)\right)}^{2}.$$

In order to model the scenario with a DL-based architecture using a fully connected neural network, the first challenge is that typical fully connected neural networks generally learn from vector inputs. However, in this case, the input is a set. The state-of-the-art methods for such set learning tasks include Deep Sets [37] and Set Transformer [38]. However, since these methods process all elements uniformly and do not leverage sequential patterns, they are not suitable for our study, where we aim to optimize the number of model parameters and time complexity. Therefore, we introduce a vector $W_{d}=\left[k_{1},k_{2},\ldots ,k_{t}\right]$, where the elements are initialized by linearly spacing them across the spectral dimension. $W_{d}$ effectively emulates the set $\left\{k_{1},k_{2},\ldots ,k_{t}\right\}$, particularly when there are no duplicate elements. As a result, we redefine the relation between $\hat{y_{i}}$ and $f_{\theta }$ as follows:

(8) $$\hat{y_{i}}=f_{\theta }\left(\left[v_{i}\left(k_{1}\right),v_{i}\left(k_{2}\right),\ldots ,v_{i}\left(k_{t}\right)\right]\right).$$

The objective function is then redefined as follows:

(9) $$\underset{\theta ,W_{d}}{\arg\;\min}\frac{1}{N}\sum_{i=1}^{N}{\left(y_{i}-f_{\theta }\left(\left[v_{i}\left(k_{1}\right),v_{i}\left(k_{2}\right),\ldots ,v_{i}\left(k_{t}\right)\right]\right)\right)}^{2}.$$

Equation (9) represents an NP-hard problem. In order to make the problem tractable for a practical solution by leveraging the sequential pattern in the hyperspectral data, we transform the discrete band indices to a continuous domain through discrete relaxation. In this approach, we allow the discrete band indices to take on continuous values. For this purpose, we define a function d(.) that maps fractional indices into the original band indices. We restrict the domain of d(·) to [0,1] to ensure that the function bijectively maps to the original indices [1,L]. The domain of d(·) can be considered as normalized band indices between 0 and 1. d(·) can be considered the denormalization function that transforms the normalized band indices back to the scale and range of the original band indices. For a normalized band index *q* to derive the corresponding original integer band index *I*, d(·) can be defined as follows:

(10) $$\begin{array}{c} I=d(q)=\lfloor q\;\cdot \;(L-1)\rfloor +1. \end{array}$$

To approximate the reflectance value for the *i*-th sample at a fractional index *q*, we define a continuous function $B_{i}\left(.\right)$ that estimates this value through piecewise linear interpolation from the vector $v_{i}$. That is,

(11) $$B_{i}\left(q\right)=v_{i}\left[d\left(q\right)\right]+\left(v_{i}\left[d\left(q\right)+1\right]-v_{i}\left[d\left(q\right)\right]\right)\;\cdot \;\left(q\;\cdot \;(L-1)-\lfloor q\;\cdot \;(L-1)\rfloor \right).$$

The continuous optimization formulation corresponding to the discrete optimization problem is expressed as follows:

(12) $$\underset{\theta ,W_{c}}{\arg\;\min}\frac{1}{N}\sum_{i=1}^{N}{\left(y_{i}-f_{\theta }\left(\left[B_{i}\left(r_{1}\right),B_{i}\left(r_{2}\right),\ldots ,B_{i}\left(r_{t}\right)\right]\right)\right)}^{2}$$

where $W_{c}=\left[r_{1},r_{2},\ldots ,r_{t}\right]$ is the vector that emulates the selected subset of normalized band indices $\left\{r_{1},r_{2},\ldots ,r_{t}\right\}$.

It is noteworthy that $B_{i}\left(\;\cdot \;\right)$ ensures that the objective function is smooth and differentiable with respect to the normalized band indices, which are the elements of $W_{c}$. This differentiability makes the problem amenable to DL-based models with gradient-based optimization. In this case, gradient descent optimization updates not only θ but also $W_{c}$, simultaneously minimizing the objective function.

At the end of a fixed number of training iterations, the vector $W^{*}$ containing the target integer indices is achieved by first denormalizing the normalized band indices in $W_{c}$ and then rounding them to the nearest integer:

(13) $$W^{*}=\left\{\mathrm{round}\left(d\left(r_{1}\right)\right),\mathrm{round}\left(d\left(r_{2}\right)\right),\ldots ,\mathrm{round}\left(d\left(r_{t}\right)\right)\right\}.$$

However, it is possible that multiple elements of $W_{c}$ may converge to a single value, leading to duplicate elements in $W^{*}$. Therefore, as a final step, by removing any duplicate values from $W^{*}$, we derive the resulting set $S^{*}$, which contains the unique target band indices in their original integer form: $I_{1},I_{2},\ldots ,I_{t^{\prime }}$, where $t^{\prime }\leq t$.

The mathematical foundation discussed in this section is primarily based on regression problems. However, the same principles are applicable to classification problems, particularly by replacing the mean squared error function with the cross-entropy loss function as the objective function.

#### 2.2.2. Time Complexity

The continuous functions $B_{i}\left(.\right)$ remain unchanged throughout the training process and, therefore, can be constructed only once. With a fixed number of training data, training iterations, and layers and nodes in the DL model, the training time depends only on the target size. Specifically, each unit of increase in target size results in an equivalent increase in the number of model parameters, thereby proportionally increasing training time.

With a fixed target size and number of training samples, the number of model parameters for regression tasks remains unchanged regardless of the dataset. However, for classification tasks, the number of weights between the last two layers increases proportionally with the number of output classes, slightly increasing the training time.

#### 2.2.3. Architecture

**Figure 2:**
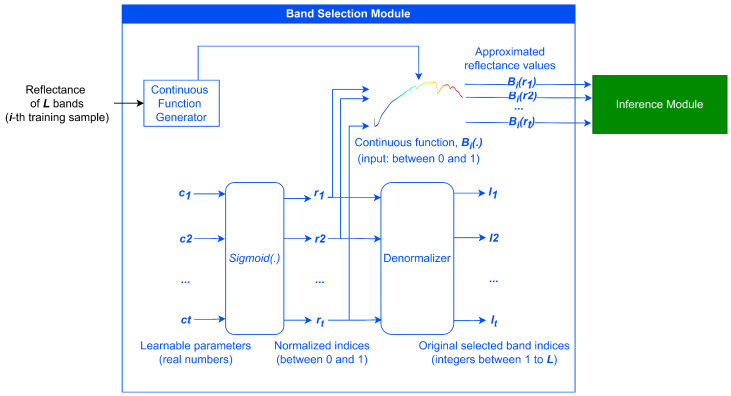
Detailed architecture of BSDR; the model parameters within the Band Selection Module ($c_{1}$, $c_{2}$, …, $c_{t}$) are initialized to correspond to indices linearly spaced across the spectral dimension, which are updated during the training process based on their predictive capability, as measured by the Inference Module.

Hyperspectral band selection algorithms can use either single pixels containing spectral information or 3D hyperspectral patches that include both spectral and spatial information as input. For clarity, we focus solely on the scenario where single pixels are used as input. The detailed architecture of BSDR is illustrated in Figure 2. First, in the Band Selection Module, we initialize a vector of learnable model parameters ($c_{1}$, $c_{2}$, …, $c_{t}$) corresponding to indices linearly spaced across the spectral dimension. These model parameters undergo a sigmoid transformation, restricting the transformed values ($r_{1}$, $r_{2}$, …, $r_{t}$) between 0 and 1, representing the normalized band indices. For the *i*-th sample, the Continuous Function Generator module produces a continuous function $B_{i}\left(\;\cdot \;\right)$, which approximates reflectance values for normalized band indices with piecewise linear interpolation as shown in the Equation (11).

**Figure 3:**
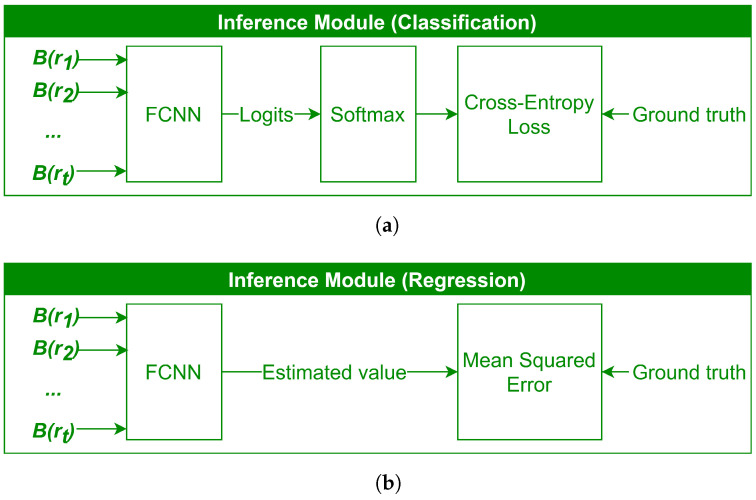
Inference Module for the following: (**a**) Classification; (**b**) Regression.

During the training process, the approximated reflectance values corresponding to the normalized band indices, denoted as $B_{i}\left(r_{1}\right),B_{i}\left(r_{2}\right),\cdots ,B_{i}\left(r_{r}\right)$, are passed as input into the Inference Module to predict the target variable. The structure of the Inference Module for classification and regression tasks has been illustrated in Figure 3a,b. Through a joint optimization process, all the learnable parameters in the entire architecture, including the weights and biases in the Inference Module and the learnable model parameters in the Band Selection Module corresponding to the target indices, are simultaneously updated.

The internal configuration of the Inference Module is designed such that the efficacy of the overall model depends more on the selected band indices than on the relational modeling capabilities of the module. Consequently, it has been deliberately simplified compared to the deeper layers used in previous studies [15,16]. Considering the available training data in the three datasets, we opted for a two-layer architecture with 128 and 64 nodes, respectively. To introduce non-linearity, we employed the Leaky ReLU activation function [39] after each hidden layer, as it is more favorable than the commonly used ReLU due to its mitigation of the dying neuron issue [40], thereby improving learning dynamics. For datasets with fewer training samples, a simpler configuration may be adopted.

After training is complete, by denormalizing these normalized indices and then rounding them to the nearest integers, the vector $W^{*}$ containing the target indices is achieved as shown in Equation (13). Finally, we derive the resulting set $S^{*}$ by removing any duplicate values from $W^{*}$.

While mini-batch training is more common in DL, in this study, we utilized full-batch training due to the significantly low number of model parameters. Training with the entire dataset to compute the gradient in each epoch ensures more accurate and stable gradient calculations. This approach mitigates the noise and variance in gradient estimates that typically occur with smaller batches.

A low number of training epochs results in suboptimal band selection, whereas excessive epochs can lead to overfitting within the Inference Module. Consequently, the selected bands become overly dependent on DL-based algorithms, diminishing their effectiveness when applied to alternative algorithms, such as SVM, within the context of this study. Following empirical observations, a balanced number of 500 training epochs has been determined.

Since the target bands are represented by learnable parameters, which are updated through the gradient descent algorithm, BSDR is highly sensitive to the learning rate. An aggressive learning rate can easily cause overshooting in band selection, while a low learning rate may fail to select an effective set of bands before training concludes. A learning rate of 0.001 has been found to be effective, offering a balanced approach.

### 2.3. Experimental Design and Objectives

For the three datasets, we aim to identify the target feature set for target sizes of 5, 10, 15, 20, 25, and 30 using all algorithms. Similar to previous studies [15,16], the efficacy of the bands selected by each algorithm is evaluated using a support vector machine (SVM) with a radial basis function kernel. As BS-Net-Classifier [15] is focused on classification, we limit its comparison to classification tasks only. For Cai et al.’s algorithm [16], we use BS-Net-FC since BS-Net-Conv is designed for 3D spectral–spatial data.

**Figure 4:**
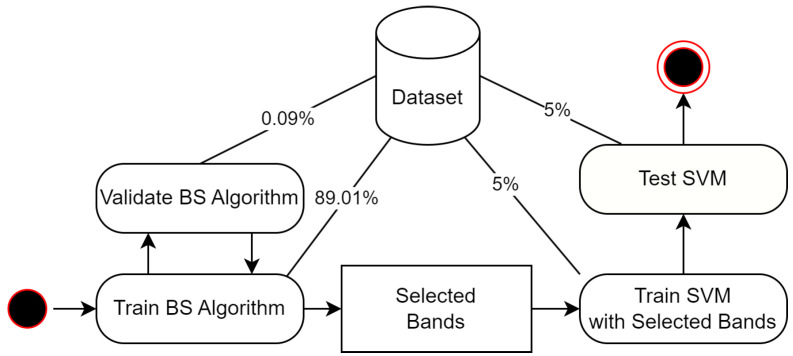
Activity diagram illustrating the experimental design, including data-split percentages for each activity: training and validating the band selection (BS) algorithm and training and testing SVM with the selected bands.

To ensure a fair and unbiased comparison of the algorithms, we apply 10-fold cross-validation. In each each cross-validation iteration, 90% of the data are randomly sampled for training the band selection algorithm, while the remaining 10% is used to evaluate the selected bands using an SVM classifier. From the training data, a randomly sampled 10% subset is allocated for model validation, with the rest used for training. Additionally, the evaluation split is randomly divided into two equal parts, one for training and the other for testing the SVM model on the selected bands. Figure 4 illustrates the process during each iteration. The prediction performance is also evaluated using all bands using SVM to assess the band selection efficacy of each algorithm relative to the performance without any data reduction. Due to the extensive time required by SPA for datasets with a large number of features, SPA was excluded from the 10-fold cross-validation for the LUCAS dataset, and only a single run was executed.

For hyperparameter selection in SVM, the regularization parameter *C* was set to 100 for regression and to $10^{5}$ for classification, respectively, while the kernel coefficient γ was set to 1 for both, all determined through grid search.

#### 2.3.1. Evaluation Metrics

To compare the performance of BSDR with other algorithms, we measure Overall Accuracy (OA) and Cohen’s Kappa (κ) for classification tasks, as performed in previous studies [15,16]. While OA provides a straightforward measure of the proportion of correctly classified instances to the total, κ offers a more nuanced assessment by considering the agreement beyond chance between predicted and actual class labels. OA is intuitive and easy to interpret but may overlook imbalances in class distribution, whereas κ adjusts for chance agreement, making it particularly valuable for assessing classifier performance in datasets with imbalanced classes or when considering the potential for chance agreement. For regression, we use $R^{2}$ and Root Mean Square Error (RMSE), which are common metrics for regression problems. $R^{2}$ gauges the proportion of variance explained by the regression model, indicating how well it fits the data, while RMSE measures the average size of prediction errors, directly reflecting model accuracy. For both classification and regression, we also measure the time required to train each band selection algorithm in seconds.

#### 2.3.2. Objectives

The specific objectives of our experiments are to compare and analyze the performance of BSDR with the benchmark algorithms as per the following aspects:

1. The number of model parameters used by each DL-based algorithm.
2. Regarding classification tasks for the algorithms MCUVE, SPA, BS-Net-FC, BS-Net-Classifier, and BSDR:
  2.1 Time efficiency.
  2.2 Predictive accuracy in terms of OA and κ.
  2.3 Changes in target band indices throughout the training process.
3. Regarding the regression task for the algorithms MCUVE, SPA, BS-Net-FC, and BSDR:
  3.1 Time efficiency.
  3.2 Estimation accuracy in terms of $R^{2}$ and RMSE.
  3.3 Changes in target band indices throughout the training process.
4. Bands selected by BSDR for each of the 10 training folds across all three datasets. For presentation purposes, only the case with a target size of five has been chosen.
5. Bands selected by BSDR for each of the 10 training folds where two or more target band indices have converged to a single value for at least one of the training folds.

## 3. Results

### 3.1. Model Parameters

To accomplish Objective 1, which is listed in Section 2.3.2, the number of model parameters used by each DL-based algorithm across various datasets is presented in Table 2. The values presented for BSDR correspond to a target size of 5. For larger target sizes, the number of required parameters increases slightly. For the other algorithms, the number of parameters does not depend on the target size. As evident from the table, BSDR requires a significantly lower number of parameters compared to the other algorithms.

### 3.2. Classification

#### 3.2.1. Time Complexity

**Figure 5:**
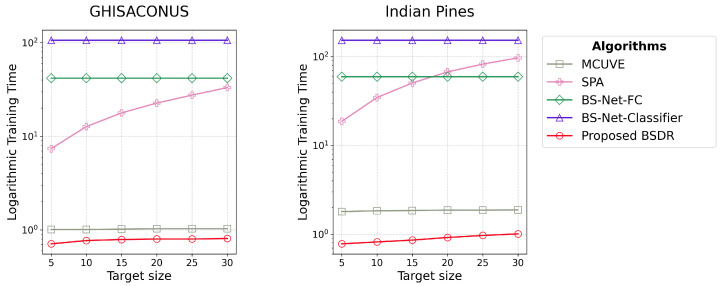
Training time (seconds) in logarithmic scale required by each band selection algorithm for the classification task for GHISACONUS and Indian Pines datasets, demonstrating the superior time efficiency of the proposed algorithm BSDR.

Figure 5 corresponds to Objective 2.1 of Section 2.3.2, which shows the training time required for each algorithm for the GHISACONUS and Indian Pines datasets. Since the scale of the training time varies greatly across the algorithms, for the convenience of illustration, the results are shown on a logarithmic scale.

The comparative differences in training time for the algorithms are consistent across the two datasets. Training time is independent of the target size for the attention-based models, BS-Net-FC [16] and BS-Net-Classifier [15]. BSDR requires significantly less training time than SPA and the attention-based algorithms. MCUVE is close to BSDR in terms of training time, although it is slightly higher.

#### 3.2.2. Predictive Accuracy

**Figure 6:**
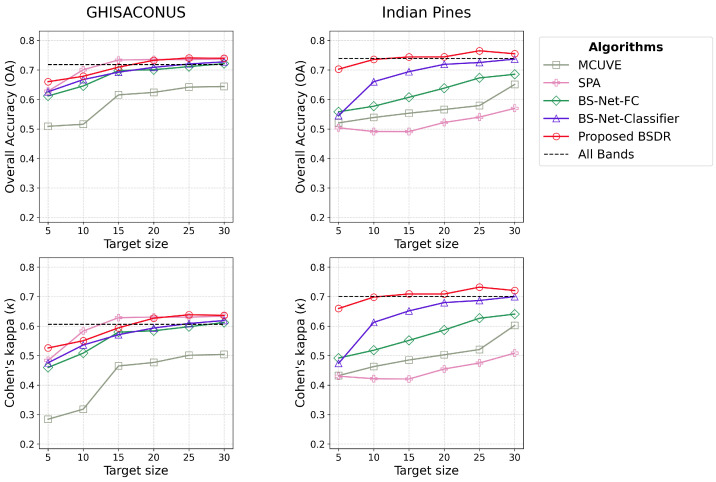
Predictive performance of the algorithms for classification tasks, shown in terms of Overall Accuracy (OA) and Cohen’s kappa (κ) for the GHISACONUS and Indian Pines datasets, demonstrating the superior accuracy of the proposed BSDR algorithm.

Objective 2.2 under Section 2.3.2 is addressed in Figure 6 and Table 3 and Table 4, which represent the predictive accuracy of the classification tasks.

Both OA and κ are consistent across the GHISACONUS and Indian Pines datasets as shown in Figure 6. Unlike the training time, however, the comparative predictive accuracy of the algorithms varies between the datasets. While SPA dominates in the GHISACONUS dataset, its performance is notably inferior in the other dataset. BSDR consistently outperforms all other algorithms in the Indian Pines dataset, with performance comparable to SPA in GHISACONUS. Notably, BSDR surpasses the performance with all bands while selecting 20 or more bands in GHISACONUS, and 15 or more bands in Indian Pines. The optimal performance is achieved with 25 bands for Indian Pines. It is also noteworthy that BS-Net-Classifier performs better than BS-Net-FC in both datasets.

Table 3 and Table 4 summarize the OA results from the 10-fold cross-validation of the algorithms for different target sizes in the GHISACONUS and Indian Pines datasets, respectively. The standard deviation for BSDR across different executions is minimal, ranging from 0.02 to 0.04. BSDR demonstrates its most outstanding comparative performance for a target size of 5 on the Indian Pines dataset, achieving an OA of 0.7. This represents a 25% improvement over the closest competitor between the two attention-based algorithms, BS-Net-FC [16], which achieved an OA of 0.56, and a 34.62% improvement over the closest competitor between the two traditional algorithms, MCUVE, which achieved an OA of 0.52.

#### 3.2.3. Band Selection Across Training Iterations

**Figure 7:**
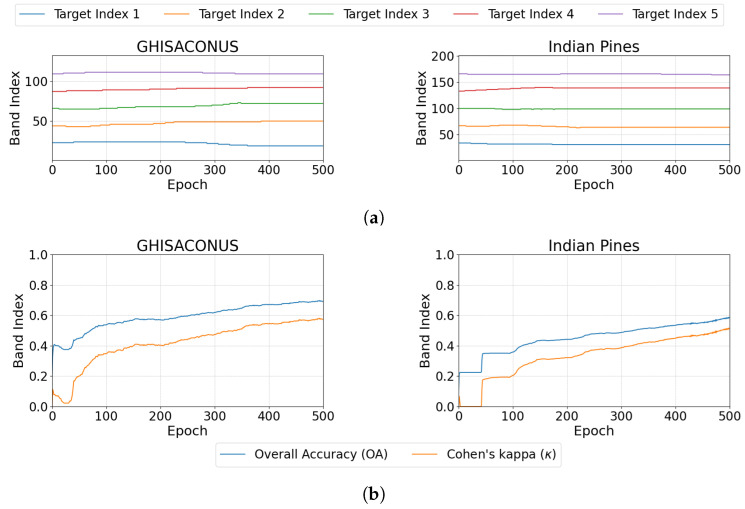
Changes in (**a**) band selection and corresponding (**b**) validation performance for two datasets across the training epochs; the validation performance improves as training progresses.

Figure 7 addresses Objective 2.3 of Section 2.3.2, illustrating the selection of important bands by BSDR throughout the training epochs for the GHISACONUS and Indian Pines datasets (Figure 7a), along with corresponding updates in the OA and κ (Figure 7b). Although the band indices undergo minor updates throughout the epochs, these cumulative changes enhance OA and κ as training progresses. These metrics have been measured within BSDR using the validation dataset described in Section 2.3 to monitor the training progress.

### 3.3. Regression

#### 3.3.1. Time Complexity

**Figure 8:**
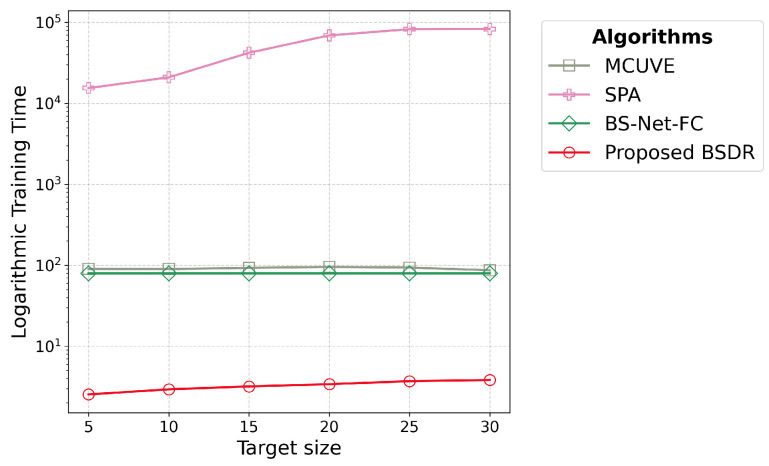
Training time (seconds) in logarithmic scale for each band selection algorithm for the regression task on the LUCAS dataset demonstrates that the proposed BSDR algorithm achieves superior time efficiency compared to the other algorithms by a significant margin.

Figure 8 and Table 5 correspond to Objective 3.1 under Section 2.3.2. Figure 8 shows the training time required by the algorithms for the regression task in the LUCAS dataset. Similar to the classification tasks, BSDR requires a significantly smaller amount of time than the other algorithms, while the time required to train SPA is excessively high. Table 5 summarizes the training time required by the algorithms for the regression task in the LUCAS dataset, based on 10-fold cross-validation. As mentioned in Section 2.3, SPA was executed only once due to its excessive training time; therefore, its stability for training time in different sets of training data has not been explored. In contrast, BSDR not only requires significantly less time than the other algorithms but also demonstrates consistent training times across different training sets, with a small standard deviation. Furthermore, as the target size increases, the training time for BSDR shows only a slight increase. BSDR exhibits its most efficient performance at a target size of 5, requiring only 2.54 s. This represents a 96.8% reduction in execution time compared to the attention-based competitor BS-Net-FC [16], which took 79.33 s, and a 97.18% reduction compared to the closest competitor between the two traditional algorithms, SPA, which took 90.02 s.

#### 3.3.2. Predictive Accuracy

**Figure 9:**
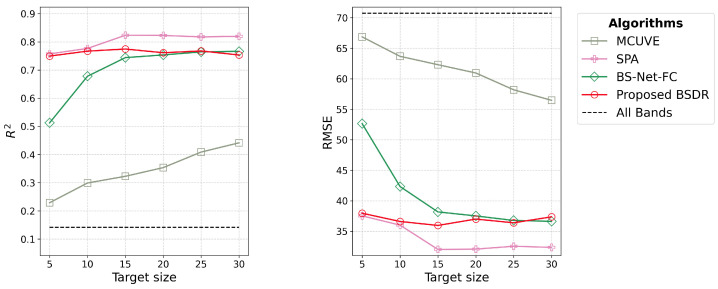
Band selection performance on the LUCAS dataset in terms of $R^{2}$ and RMSE for different algorithms; using all 4200 bands results in significantly inferior performance, as the evaluation algorithm (SVM) struggles to model the relationship between 4200 bands and the response variable (SOC) with only 1089 training samples.

Objective 3.2 under Section 2.3.2 is addressed by Figure 9 and Table 6. Figure 9 shows the estimation performance of the algorithms for the regression task in the LUCAS dataset. SPA outperforms the other algorithms in terms of accuracy. For BSDR, performance begins to deteriorate when selecting more than 15 bands and gradually converges with that of BS-Net-FC. Unlike in the GHISACONUS and Indian Pines datasets, the estimation performance with all bands is significantly lower in the LUCAS dataset. Based on the results measured from a single run of SPA, Table 6 shows that SPA outperforms the other algorithms for the regression task in the LUCAS dataset. BSDR maintains a low standard deviation ranging from 0.03 to 0.06 across the 10-fold validation, while remaining comparable to SPA.

#### 3.3.3. Band Selection Across Training Iterations

**Figure 10:**
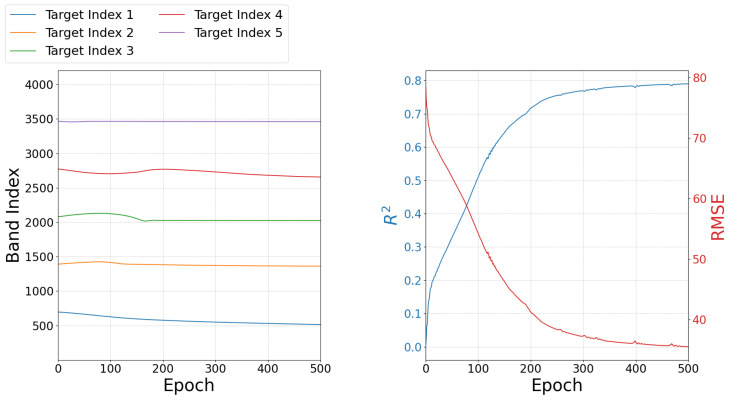
Changes in band selection and corresponding validation performance for regression across training epochs; the performance improves as the training progresses.

In reference to Objective 3.3 in Section 2.3.2, Figure 10 illustrates the update of the selected bands by BSDR during training, along with the corresponding validation performance. Similar to the classification tasks, performance improves as the combination of bands is updated, despite the band indices undergoing minor updates throughout the training. The metrics $R^{2}$ and RMSE have been measured internally within BSDR with the validation data.

### 3.4. Selected Bands

Table 7 illustrates the bands selected in a sorted order by BSDR for each training fold in three datasets, specifically for a target size of five, as specified in Objective 4 listed in Section 2.3.2. The selected bands remain stable across the training folds with some minor differences due to the randomness introduced by the training folds. For example, in GHISACONUS, while the first band is always 19, and the most common indices for the other positions are 43, 63, 91, and 110, respectively, they slightly change in some training folds.

Table 8 addresses Objective 5 in Section 2.3.2, which lists the bands selected by BSDR for each of the 10 training folds where two or more target band indices have converged to a single value for at least one of the training folds. This figure corresponds to the case GHISACONUS dataset with a target size of 10. In this case, 10 bands have been selected in most of the cases as specified by the target size, and the selected bands remain stable across the training sets with some minor differences, as shown in Table 7. However, in one instance, for the ninth fold, nine bands were selected instead of 10, meaning two of the target indices converged to a single value.

## 4. Discussions

### 4.1. Model Parameters

For BSDR, the Band Selection Module’s model parameters correspond to the target size. In the Inference Module, this count remains unchanged for regression tasks. For classification tasks, the weights between the last two linear layers slightly increase as the number of target classes increases. As a result, the number of model parameters increases slightly, from 9354 to 10,069, for GHISACONUS and Indian Pines, respectively, as shown in Table 2.

Both BS-Net-FC [16] and BS-Net-Classifier [15] utilize deep and wide layers, resulting in a significantly higher number of model parameters compared to BSDR. Furthermore, for both algorithms, the model parameter count increases linearly with the number of bands. The implication becomes evident when considering the significant number of model parameters, as large as 2,226,416, required by BS-Net-FC for the LUCAS dataset, consisting of 4200 bands. This is 14 and 18 times more than the number of parameters required by BS-Net-FC for Indian Pines and GHISACONUS, respectively, each comprising only a few hundred bands.

### 4.2. Classification

#### 4.2.1. Time Complexity

The superior time efficiency depicted in Figure 5 stems from BSDR’s use of full-batch training and its reliance on a minimal number of parameters compared to other deep learning-based algorithms. The modest increase in training time for BSDR with the target size is attributable to the corresponding slight increase in model parameters, as discussed in Section 3.1. BS-Net-Classifier requires 500 training epochs, compared to BS-Net-FC’s 100 epochs. Consequently, it takes more time to train than BS-Net-FC. Training time for BS-Net-FC and BS-Net-Classifier remain constant across different target sizes, as these models attempt to derive weights for each band regardless of the target size. Since SPA selects new bands iteratively based on their information content, projecting them into orthogonal spaces through complex calculations, the training time escalates with the target size. This increase in training time is attributed to the growing number of iterations required as more bands are considered. Since MCUVE iteratively eliminates uninformative bands through a randomized approach and does not consider extensive combinations of features like SPA does, it requires less time to train than SPA.

#### 4.2.2. Predictive Accuracy

In terms of predictive accuracy on the classification tasks, the closest competitor to BSDR is SPA, as shown in Figure 6. While BSDR consistently surpasses SPA on the Indian Pines dataset, it trails SPA slightly on the GHISACONUS dataset for a few target sizes. This can be attributed to the consecutive missing bands across different regions. BSDR mistakenly treats two consecutive bands as adjacent, even when a large number of bands are missing in between, which leads to its degraded performance on the GHISACONUS dataset. However, the limited applicability of SPA is evident in the Indian Pines dataset, where it exhibits the most inferior performance. The disparity in SPA’s performance between the two datasets arises because SPA selects informative bands in an unsupervised fashion without considering the target task. Given that the Indian Pines dataset contains more classes than GHISACONUS, 16 compared to five, SPA struggles to maintain consistent performance as the increased complexity from a greater number of classes hampers its ability to effectively discriminate among them. This rationale also explains why BS-Net-Classifier outperforms BS-Net-FC, especially on the more complex task on the Indian Pines dataset, which has a higher number of target classes. BS-Net-Classifier is tailored specifically to the classification task, fully utilizing training data to optimize performance. In contrast, BS-Net-FC learns band importance based on their capability to reconstruct the data in an unsupervised fashion without any information about the target task. Additionally, the superiority of BS-Net-Classifier is further enhanced by a sparse layer they utilized, which eliminates bands falling below a certain weight threshold, eliminating the uninformative bands concerning the target task.

The observed divergence between κ and OA for BS-Net-Classifier model, noted for the smaller target size of five in the Indian Pines dataset, suggests that the high OA may be more attributable to chance agreement rather than the true predictive efficacy of the algorithm for that instance. This divergence may stem from the large number of parameters in the model, which may induce overfitting. Additionally, a comparatively higher standard deviation in OA for target size five, reported as 0.06 in Table 4, further supports this observation.

For GHISACONUS, BS-Net-Classifier and BS-Net-FC require 25 and 30 bands, respectively, to achieve the OA attained using all bands. In contrast, BSDR surpasses the OA attained with all bands by utilizing only 20 bands. Additionally, the consistent κ for supports the true predictive accuracy of BSDR, indicating its effectiveness is not due to chance agreement.

In the Indian Pines dataset, BS-Net-Classifier and BSDR require 30 and 10 bands, respectively, to achieve the OA attained using all bands. Similarly, the consistent κ for BSDR at the target size of 10 supports the interpretation that its results reflect a true predictive capability rather than chance agreement.

In both datasets, BSDR achieves the best results with a target size of 25; however, beyond this point, its performance begins to deteriorate. The small standard deviation of the OA results for BSDR shown in Table 3 and Table 4, based on the 10-fold cross-validation, supports the reliability of the presented results across various training data splits.

#### 4.2.3. Band Selection Across Training Iterations

Throughout the training epochs, the selected band indices are updated, as illustrated in Figure 7. BSDR shows sensitivity to the initial values of the target indices, often converging toward a local optimum near the initial position. However, since many hyperspectral bands are linearly correlated, meaning that several bands can be reliably constructed as combinations of one or more bands, an effective combination can be achieved with small adjustments to each target band index, as demonstrated by BSDR.

As Figure 7 presents, the validation performance maintains a positive direction till the end of the training. It may seem prudent to continue training for more epochs. However, we observed that extended training contributes more significantly to the Inference Module. Beyond a certain point, gradient updates do not effectively propagate to the earlier layers, where the learnable band indices are located. Additionally, overtraining the Inference Module may result in diminished efficacy of the selected bands when using algorithms that do not employ DL.

### 4.3. Regression

#### 4.3.1. Time Complexity

As mentioned in the Introduction, BS-Net-Classifier has been excluded from the regression task as it is exclusively designed for classification. Among the other algorithms, BSDR demonstrates the highest time efficiency, as shown in Figure 8 and Table 5, similar to the classification tasks. Even with a high spectral dimension of 4200 bands, the training time remains low because BSDR processes only a limited number of bands matching the target size, regardless of the spectral dimension size. The low standard deviation in execution time across multiple runs shown in Table 5 highlights the reliability of BSDR’s superior time efficiency. The limitations of SPA become more pronounced with the LUCAS dataset. While the training time for SPA remained comparable to other algorithms for datasets containing a few hundred bands, as shown in Figure 5, it increased disproportionately for the LUCAS dataset. This raises questions about its applicability in high-dimensional datasets. For the same rationale presented in Section 4.2 regarding the random selection approach of MCUVE, the training time required for it remains within a comparable range with the other algorithms despite the high spectral dimension. Training time remains constant for BS-Net-FC as it evaluates weights for all the bands regardless of the target size.

#### 4.3.2. Predictive Accuracy

As shown by the dashed lines in Figure 9, the performance on the LUCAS dataset using all bands is significantly lower than in the classification tasks with the GHISACONUS and Indian Pines datasets. This is primarily due to the much higher spectral dimension of the LUCAS dataset compared to the other two. Notably, an $R^{2}$ value of up to 0.91 has been reported in the literature [41] for SOC estimation from the LUCAS dataset using SVM. This result was achieved through rigorous preprocessing, outlier removal, and the exclusion of certain bands based on domain knowledge. Additionally, 14,277 training samples were used, whereas in our study, only 5% of the samples (1089) were used for training. Most notably, their approach involved downsampling the data by retaining one band every 10 nm, resulting in a reduced set of 195 bands.

Without such dimensionality reduction techniques, either through downsampling and band removal based on domain knowledge or automatic band selection using a suitable algorithm, many machine learning models may struggle to perform on high-dimensional datasets like LUCAS. Therefore, better estimation accuracy can be achieved even with five bands selected by the lowest-performing algorithm, MCUVE. These findings underscore the critical importance of dimensionality reduction, particularly for datasets with a high degree of collinearity or redundant features.

SPA performs consistently better than the other algorithms across different target sizes. However, this performance comes at the cost of a disproportionately long training time, ranging from 1.54 × 10^4^ to 8.88 × 10^4^ s based on the target size, as presented in Table 5. The performance of BSDR consistently improves across target sizes up to 15; beyond this point, it either deteriorates or remains unchanged at specific intervals. This deterioration could be attributed to the high degree of collinearity in the high-resolution visible and near-infrared spectra [42] provided by the LUCAS dataset. With as many as 4200 bands in the dataset, evaluating a large number of band combinations is crucial, as SPA demonstrates, to achieve effective band selection for target sizes exceeding 15.

The low standard deviation of the $R^{2}$ values for BSDR compared to BS-Net-FC up to target size 20, as presented in Table 6 based on 10-fold cross-validation, illustrates the consistent performance of the BSDR model across different training sets. Since SPA was executed only once due to its excessive training time, the standard deviation of its performance measures could not be determined.

#### 4.3.3. Band Selection Across Training Iterations

Similar to classification tasks, significant changes in validation $R^{2}$ and RMSE are observed with minor updates in the learnable band indices as training progresses for the regression task, as illustrated in Figure 10. The consistent increase in validation $R^{2}$ and decrease in validation RMSE suggest that the model is not only fitting effectively to the data but also generalizing effectively to unseen data. This trend implies a successful capture of the underlying patterns rather than memorization of the training set, as supported by the evaluated performance presented in Table 6.

### 4.4. Analysis of Band Selection Stability

The introduction of randomness by the training sets subtly influences the learning process of BSDR. These effects are manifested in the slight variations in band selection for certain indices, as demonstrated in Table 7 and Table 8. The differences are comparatively bigger in LUCAS as the gradient-based search occurs through a higher spectral dimension, consisting of 4200 bands, in contrast to a few hundred bands in the other datasets.

**Figure 11:**
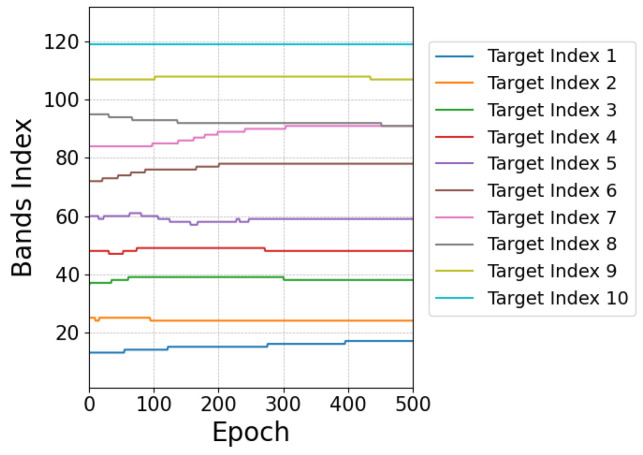
Example of multiple target indices converging to a single value, resulting in fewer selected bands than the specified target size. In this training case, toward the end of training, both the seventh and eighth target indices assume the value 91.

As mentioned in Section 2.2, duplicate bands are removed after training concludes. Consequently, the number of selected bands might be lower than the intended target size in some instances. This is exemplified by the ninth training fold for the GHISACONUS dataset, where nine bands were selected, although the specified target size was 10, as shown in Table 8. The investigation through that particular instance of training reveals that after the 452nd epoch, both the seventh and eighth target indices assume the value of 91, which remains unchanged until the end of the training, as illustrated in Figure 11. The selected set was ultimately reduced to nine bands after removing the duplicate value of band 91.

### 4.5. Overall Analysis

From Figure 6 and Figure 9, it is evident that, except for some specific cases, BSDR consistently outperforms attention-based algorithms in predictive accuracy. This superior performance can be attributed not only to the data efficiency of BSDR, which stems from its reduced number of model parameters, but also to a fundamental limitation of ranking-based algorithms like BS-Net-FC [16] and BS-Net-Classifier [43]. While ranking-based methods provide a robust mechanism for ranking bands by importance, they fail to account for intraband correlations [44], often leading to a subset of bands with higher information redundancy. Moreover, some low-ranked bands that may exhibit strong correlations with the target variable when combined with other bands [45] are likely to be excluded.

In contrast, BSDR dynamically updates the selected bands based on their collective influence, thereby more effectively capturing intraband correlations. This consideration is particularly crucial for smaller target sizes, where redundancy reduces the proportion of informative bands, resulting in a more pronounced decline in predictive accuracy. The efficacy of BSDR in accounting for intraband correlations is demonstrated in Figure 6 and Figure 9, as evidenced by the larger performance gap at lower target sizes. Across all three datasets, the performance gap between BSDR and the two attention-based algorithms is most pronounced at the lowest target size, 5.

## 5. Conclusions

This study demonstrates that the proposed model, BSDR, effectively selects hyperspectral bands, using a gradient-based search across the spectral dimension, as evidenced by a comprehensive evaluation of three benchmark datasets, which encompass diverse geographic regions, significant variability in soil types, and challenges such as class imbalance. By transforming discrete integer band indices into real numbers through discrete relaxation, BSDR reduces the NP-hard problem of band selection to polynomial time concerning the specified target size, providing a practical solution. Furthermore, this study shows that piecewise linear interpolation can effectively approximate the reflectance values for the fractional indices used in the gradient-based optimization.

Since BSDR processes only a limited number of bands matching the target size, regardless of the original spectral dimension, it requires significantly fewer model parameters. Consequently, it is not only highly data-efficient but also requires noticeably less training time compared to other recent DL-based band selection algorithms. This approach also allows BSDR to accommodate a high spectral dimension with as many as 4200 bands without requiring significantly more training time, which is unlike other comparably accurate algorithms such as SPA. Furthermore, BSDR demonstrated an effective approach to subset selection by learning through vector input and regulating the vector to closely emulate a set.

A limitation observed in BSDR is its high sensitivity to parameter initialization, which occasionally causes it to converge to a local optimum solution. In future studies, we intend to address this limitation by employing multiple initializations in parallel and deriving a combined decision based on them. Additionally, we will aim to develop a solution that addresses the issue of missing bands, thus ensuring that BSDR correctly distinguishes between adjacent and non-adjacent bands to improve performance on datasets like GHISACONUS.

## Figures and Tables

**Table 1:** Details of the datasets used in this study.

Dataset	Task	Target Variable	Samples	Bands
GHISACONUS	Classification	16 classes	6933	131
Indian Pines		5 crops	10,249	200
LUCAS	Regression	SOC content	21,782	4200

**Table 2:** Number of model parameters used by each DL-based band selection algorithm for the three datasets; BSDR requires substantially fewer model parameters than the other algorithms.

Algorithm	GHISACONUS	Indian Pines	LUCAS
BS-Net-FC [16]	118,674	154,416	2,226,416
BS-Net-Classifier [15]	135,724	207,740	N/A
Proposed BSDR	9354	10,069	9094

**Table 3:** Overall Accuracy (OA) based on 10-fold cross-validation for different target sizes on the GHISACONUS dataset, with the best results in bold and the second best in blue and italicized, showing the proposed BSDR algorithm achieves the best or comparable OA.

	Target Size					
**Algorithm**	**5**	**10**	**15**	**20**	**25**	**30**
MCUVE [13]	0.51 ± 0.05	0.52 ± 0.03	0.62 ± 0.04	0.62 ± 0.03	0.64 ± 0.04	0.64 ± 0.04
SPA [14]	*0.63 ± 0.03*	**0.70 ± 0.03**	**0.73 ± 0.03**	**0.74 ± 0.03**	**0.74 ± 0.03**	**0.74 ± 0.04**
BS-Net-FC [16]	0.61 ± 0.03	0.65 ± 0.03	0.70 ± 0.03	0.70 ± 0.02	0.71 ± 0.03	0.72 ± 0.03
BS-Net-Classifier [15]	*0.63 ± 0.03*	0.67 ± 0.04	0.69 ± 0.04	0.71 ± 0.03	*0.72 ± 0.04*	*0.73 ± 0.04*
Proposed BSDR	**0.66 ± 0.03**	*0.68 ± 0.03*	*0.71 ± 0.03*	*0.73 ± 0.03*	**0.74 ± 0.04**	**0.74 ± 0.03**

**Table 4:** Overall Accuracy (OA) based on 10-fold cross-validation for different target sizes on the Indian Pines dataset, with the best results in bold and the second best in blue and italicized, demonstrating that the proposed BSDR algorithm outperforms other algorithms by a noticeable margin.

	Target Size					
**Algorithm**	**5**	**10**	**15**	**20**	**25**	**30**
MCUVE [13]	0.52 ± 0.04	0.54 ± 0.05	0.55 ± 0.03	0.57 ± 0.03	0.58 ± 0.02	0.65 ± 0.04
SPA [14]	0.50 ± 0.03	0.49 ± 0.03	0.49 ± 0.02	0.52 ± 0.02	0.54 ± 0.02	0.57 ± 0.03
BS-Net-FC [16]	*0.56 ± 0.02*	0.58 ± 0.02	0.61 ± 0.03	0.64 ± 0.03	0.67 ± 0.02	0.69 ± 0.02
BS-Net-Classifier [15]	0.55 ± 0.06	*0.66 ± 0.05*	*0.69 ± 0.03*	*0.72 ± 0.03*	*0.73 ± 0.03*	*0.74 ± 0.03*
Proposed BSDR	**0.70 ± 0.04**	**0.74 ± 0.03**	**0.74 ± 0.02**	**0.74 ± 0.03**	**0.77 ± 0.02**	**0.75 ± 0.02**

**Table 5:** Training time in seconds based on 10-fold cross-validation results for different target sizes on the LUCAS dataset, with the best results in bold and the second best in blue and italicized; the small standard deviation across different executions for the proposed BSDR algorithm demonstrates the reliability of its superior time efficiency.

	Target Size					
**Algorithm**	**5**	**10**	**15**	**20**	**25**	**30**
MCUVE [13]	90.02 ± 1.67	89.90 ± 1.81	93.02 ± 2.89	95.50 ± 22.95	93.81 ± 10.00	86.85 ± 1.76
SPA [14] ^1^	1.54 × 10^4^	2.10 × 10^4^	4.22 × 10^4^	6.91 × 10^4^	8.23 × 10^4^	8.88 × 10^4^
BS-Net-FC [16]	*79.33 ± 1.54*	*79.33 ± 1.54*	*79.33 ± 1.54*	*79.33 ± 1.54*	*79.33 ± 1.54*	*79.33 ± 1.54*
Proposed BSDR	**2.54 ± 0.02**	**2.94 ± 0.01**	**3.19 ± 0.01**	**3.40 ± 0.03**	**3.71 ± 0.02**	**3.83 ± 0.02**

^1^ Since SPA was executed only once due to its excessive training time, the standard deviation of its execution time could not be determined.

**Table 6:** $R^{2}$ based on 10-fold cross-validation results for different target sizes on the LUCAS dataset; the best (bold) and the second best (blue and italicized) results are highlighted.

	Target Size					
**Algorithm**	**5**	**10**	**15**	**20**	**25**	**30**
MCUVE [13]	0.23 ± 0.17	0.30 ± 0.08	0.32 ± 0.11	0.35 ± 0.12	0.41 ± 0.09	0.44 ± 0.11
SPA [14] ^1^	**0.76**	**0.78**	**0.82**	**0.82**	**0.82**	**0.82**
BS-Net-FC [16]	0.51 ± 0.11	0.68 ± 0.11	0.74 ± 0.05	0.75 ± 0.05	0.76 ± 0.04	*0.77 ± 0.03*
Proposed BSDR	*0.75 ± 0.03*	*0.77 ± 0.03*	*0.77 ± 0.04*	*0.76 ± 0.04*	*0.77 ± 0.05*	0.75 ± 0.06

^1^ Since SPA was executed only once due to its excessive training time, the standard deviation of $R^{2}$ for this could not be determined.

**Table 7:** Bands selected by BSDR across different training folds for three datasets at target size five; the selected bands remain stable with minor changes in some cases.

	Dataset		
**Fold**	**GHISACONUS**	**Indian Pines**	**LUCAS**
1	19, 50, 72, 92, 109	31, 64, 99, 139, 164	515, 1362, 2024, 2657, 3460
2	19, 43, 63, 91, 110	31, 64, 99, 141, 165	515, 1399, 2024, 2651, 3462
3	19, 44, 63, 91, 110	31, 64, 99, 141, 165	522, 1228, 2024, 2694, 3460
4	19, 44, 63, 91, 110	31, 64, 99, 139, 164	515, 1345, 2024, 2690, 3461
5	19, 44, 63, 91, 110	31, 64, 99, 140, 164	516, 1370, 2024, 2670, 3460
6	19, 43, 63, 91, 110	31, 64, 99, 141, 165	518, 1300, 2025, 2692, 3461
7	19, 43, 63, 91, 110	31, 64, 99, 140, 164	524, 1223, 2024, 2747, 3462
8	19, 43, 63, 91, 110	31, 64, 99, 141, 164	517, 1390, 2024, 2667, 3462
9	19, 43, 63, 91, 110	31, 64, 99, 141, 164	517, 1399, 2024, 2653, 3461
10	19, 44, 63, 91, 110	32, 64, 99, 140, 164	519, 1318, 2024, 2749, 3462

**Table 8:** Bands selected by BSDR across different training folds for the GHISACONUS dataset at a target size of 10; the ninth training fold demonstrates an example of achieving a lower number of bands than specified.

Fold	GHISACONUS
1	18, 24, 38, 48, 59, 78, 90, 92, 107, 119
2	17, 24, 38, 48, 59, 78, 91, 92, 109, 118
3	17, 24, 38, 48, 59, 78, 91, 92, 108, 118
4	17, 24, 38, 48, 59, 78, 91, 92, 109, 119
5	17, 24, 37, 48, 59, 78, 90, 92, 108, 119
6	17, 24, 38, 48, 59, 78, 90, 91, 108, 119
7	17, 24, 38, 48, 59, 78, 91, 92, 108, 119
8	17, 24, 38, 48, 59, 78, 90, 92, 107, 119
9	17, 24, 38, 48, 59, 78, 91, 107, 119
10	17, 24, 37, 48, 59, 78, 91, 92, 107, 119

## Data Availability

The data presented in this study are available in the NASA EOSDIS Land Processes DAAC at https://doi.org/10.5067/Community/GHISA/GHISACONUS.001, reference number GHISACONUS.001, in the Purdue University Research Repository at https://doi.org/10.4231/R7RX991C, reference number R7RX991C, and in the European Soil Data Centre at https://esdac.jrc.ec.europa.eu/content/lucas-2009-topsoil-data accessed on 27 November 2024, reference number LUCAS. These data were derived from the following resources available in the public domain: https://e4ftl01.cr.usgs.gov/COMMUNITY/GHISACONUS.001/2008.01.01/GHISACONUS_2008_001_speclib.csv accessed on 27 November 2024, https://engineering.purdue.edu/~biehl/MultiSpec/hyperspectral.html accessed on 27 November 2024, and https://esdac.jrc.ec.europa.eu/content/lucas-2009-topsoil-data accessed on 27 November 2024.
